# Sub-localisation of novel proteins with unique dynamics during assembly and maintenance of the eukaryotic flagellum

**DOI:** 10.1186/2046-2530-1-S1-P13

**Published:** 2012-11-16

**Authors:** P Bastin, D Julkowska, I Subota, L Vincensini, T Blisnick, J Buisson, S Perrot, N Reeg, M Engstler

**Affiliations:** 1 Institut Pasteur, France; 2 University of Wurzburg, Germany

## 

Cilia and flagella are complex organelles composed of up to 500 proteins. We have purified intact flagella from the model organism *Trypanosoma brucei* using mechanical shearing. Scanning and transmission electron microscopy confirmed the quality and the purity of flagella and biochemical analysis demonstrated a 15-fold enrichment of flagellar markers. Mass spectrometry investigation carried out on 5 separate experiments led to the identification of 387 proteins, 55 of which had never reported to be associated to the flagellum. 10 out of the 12 proteins investigated experimentally were indeed associated to the flagellum but turned out to localise to several sub-localisations with unique profiles: flagellar membrane, axoneme, paraflagellar rod (an extra-axonemal structure) and the adhesion zone. Two of them, termed FLAMM6 and FLAMM8 showed restricted distribution to the proximal part and to the far distal end of the axoneme, respectively. Dynamics analysis revealed that membrane proteins were incorporated by the proximal end and showed a rapid turnover whereas axonemal and PFR proteins were added to the distal end of elongating flagella but showed stable association to their structure. FLAMM6 was found only in the first half of the flagellum no matter its length, a process dependent on IFT. Finally, FLAMM8 was progressively incorporated to the elongating axoneme accumulating at the distal tip where it showed very slow turnover after flagellum formation was complete. These data highlight the existence of specific micro-domains within the eukaryotic flagellum, each with its own dynamics for assembly and turnover.

